# Enhancing post-traumatic stress disorder patient assessment: leveraging natural language processing for research of domain criteria identification using electronic medical records

**DOI:** 10.1186/s12911-024-02554-8

**Published:** 2024-06-04

**Authors:** Oshin Miranda, Sophie Marie Kiehl, Xiguang Qi, M. Daniel Brannock, Thomas Kosten, Neal David Ryan, Levent Kirisci, Yanshan Wang, LiRong Wang

**Affiliations:** 1 Computational Chemical Genomics Screening Center, Department of Pharmaceutical Sciences, School of Pharmacy, University of Pittsburgh, Pittsburgh, PA 15213 USA; 2 Colorado State University, Fort Collins, CO 80521 USA; 3 RTI International, Durham, NC 27709 USA; 4 Menninger Department of Psychiatry, Baylor College of Medicine, Houston, TX 77030 USA; 5 Department of Psychiatry, School of Medicine, University of Pittsburgh, Pittsburgh, PA 15213 USA; 6 University of Pittsburgh School of Pharmacy, Pittsburgh, PA 15213 USA; 7 University of Pittsburgh School of Health and Rehabilitation Sciences, Pittsburgh, PA 15213 USA

**Keywords:** Post-traumatic stress disorder, Research of domain criteria, Real-world evidence, Clinical notes, Natural language processing

## Abstract

**Background:**

Extracting research of domain criteria (RDoC) from high-risk populations like those with post-traumatic stress disorder (PTSD) is crucial for positive mental health improvements and policy enhancements. The intricacies of collecting, integrating, and effectively leveraging clinical notes for this purpose introduce complexities.

**Methods:**

In our study, we created a natural language processing (NLP) workflow to analyze electronic medical record (EMR) data and identify and extract research of domain criteria using a pre-trained transformer-based natural language model, all-mpnet-base-v2. We subsequently built dictionaries from 100,000 clinical notes and analyzed 5.67 million clinical notes from 38,807 PTSD patients from the University of Pittsburgh Medical Center. Subsequently, we showcased the significance of our approach by extracting and visualizing RDoC information in two use cases: (i) across multiple patient populations and (ii) throughout various disease trajectories.

**Results:**

The sentence transformer model demonstrated high F1 macro scores across all RDoC domains, achieving the highest performance with a cosine similarity threshold value of 0.3. This ensured an F1 score of at least 80% across all RDoC domains. The study revealed consistent reductions in all six RDoC domains among PTSD patients after psychotherapy. We found that 60.6% of PTSD women have at least one abnormal instance of the six RDoC domains as compared to PTSD men (51.3%), with 45.1% of PTSD women with higher levels of sensorimotor disturbances compared to men (41.3%). We also found that 57.3% of PTSD patients have at least one abnormal instance of the six RDoC domains based on our records. Also, veterans had the higher abnormalities of negative and positive valence systems (60% and 51.9% of veterans respectively) compared to non-veterans (59.1% and 49.2% respectively). The domains following first diagnoses of PTSD were associated with heightened cue reactivity to trauma, suicide, alcohol, and substance consumption.

**Conclusions:**

The findings provide initial insights into RDoC functioning in different populations and disease trajectories. Natural language processing proves valuable for capturing real-time, context dependent RDoC instances from extensive clinical notes.

**Supplementary Information:**

The online version contains supplementary material available at 10.1186/s12911-024-02554-8.

## Background

Post traumatic stress disorder (PTSD) is a prevalent mental health condition affecting 6.8–7.8% of the general US population, with higher rates in areas of civil unrest or armed conflict [1]. It is characterized by disabling symptoms that often persist, leading to significant impairment in economic and social functioning and an increased risk of mortality [2]. The disorder, as defined by Diagnostic and Statistical Manual of Mental Disorders (DSM-5), encompasses symptom clusters such as hyperarousal, persistent re-experiencing of trauma, avoidance of trauma-related stimuli, and negative alterations in cognition and mood. Empirical findings reveal heterogeneity within PTSD, with subtypes characterized by hyperarousal or dissociation [3]. Untreated PTSD patients face an elevated risk of suicide-related events, substance use disorders, and other neuropsychiatric disorders.

Comorbidities exacerbate challenges, compromising social adjustments, treatment outcomes, and increasing the likelihood of early treatment termination [4]. Current approaches involve targeting individual conditions either concurrently or sequentially due to a lack of specific evidence-based interventions. Despite some interventions showing effectiveness, a substantial proportion of individuals with PTSD experience limited improvement, necessitating exploration of new, theory-based treatment options [5].

Disruptions in economic and social behaviors are significant sequelae of PTSD, leading to marital and parenting problems, high rates of comorbid disorders, unemployment, homelessness, and imprisonment [6]. The relationship between PTSD and comorbid disorders is complex and bidirectional. Traumatic stress is recognized as a vulnerability factor for substance use disorder (SUD), with a substantial proportion of SUD patients meeting PTSD criteria [7].

Artificial intelligence in mental health research is reshaping current practices. In 2010, Insel et al. initiated the RDoC project, introducing a research framework aiming to offer an alternative to the DSM. Unlike the DSM, which relies solely on clinical data, the RDoC incorporates genetics and neuroscience into its classification of mental health disorders [8]. The RDoC framework comprises a matrix featuring constructs and subconstructs across six domains: negative valence, positive valence, cognitive systems, systems for social processes, arousal or regulatory systems, and sensorimotor systems. Advocates for RDoC argue that DSM syndromes have notable limitations in identifying biomarkers and specific genetic variants associated with mental disorders [9]. One tangible application of this paradigm shift involved using statistical NLP methods to create phenotypically homogeneous cohorts for improved comparison. In 2016, the CEGS N-GRID proposed three challenging NLP tasks, including data anonymization, predicting symptom severity in the positive valence domain from neuropsychiatric clinical records, and novel data use cases such as predicting common mental conditions [10]. Despite only one study addressing the third task done in 2017 i.e. second track of the CEGS N-GRID 2016 NLP shared tasks focused on predicting symptom severity from neuropsychiatric clinical records, research on NLP and ML processing has identified several articles that met these challenges [11–16]. This marked the first instance where initial psychiatric evaluation records were collected, de-identified, annotated, and shared with the scientific community. Twenty-four teams comprising 110 researchers participated, submitting a total of sixty-five system runs for evaluation. The top ten teams achieved an inverse normalized macro-averaged mean absolute error score exceeding 0.80. The highest-performing system utilized an ensemble of six machine learning-based classifiers, achieving a score of 0.86. While the task was generally straightforward, challenges arose with records containing sparse yet critical positive valence systems, and those describing patients primarily affected by negative valence systems. These cases posed significant difficulties due to a lack of consideration of other RDoC domains, context-dependent extraction, and analysis done on small datasets, indicating the need for further research to consider the task fully solved. Overall, while these results highlight the efficacy of data-driven approaches in symptom severity classification tasks, it would be essential to consider the other domains of the RDoC framework for potential advancements in psychiatric nosology.

Recent research indicates that PTSD is linked to a diverse range of multimodal risk factors [17]. Predictive methods for PTSD using electronic medical records (EMR) data need to accommodate various combinations of risk indicators. The application of computational methods and machine learning to health data holds promise for advancing our understanding of health conditions. This study aims to characterize PTSD by leveraging the knowledge of the RDoC framework using unstructured EMR data (e.g., clinical notes). Clinical notes are crucial in healthcare, providing a detailed patient narrative that goes beyond structured data to capture nuanced information. They ensure continuity of care by documenting the patient’s journey over time, enabling tracking of conditions and adjustments to treatment plans. These notes offer insights into disease patterns and treatment effectiveness. They aid in diagnostics by capturing subjective and objective symptom information, assisting in accurate diagnoses and treatment planning. Additionally, clinical notes consider both biomedical and psychosocial factors, which is key in addressing each patient’s unique needs and preferences. Our novel framework incorporates keyword dictionaries, context-specific sentence dictionaries, the application of sentence transformer model, and identification of RDoC domains in two distinct use cases. By leveraging unstructured “big data,” this research represents a crucial step towards integrating the RDoC framework into treatment research for comorbid conditions, offering insights into etiology and treatment responses.

## Methods

### Data source

We obtained data from the Neptune system which is a clinical data warehouse at UPMC (January 2004 – October 2020). The database includes demographics, diagnoses, prescriptions, and test results. This study utilized 5.67 million clinical notes from PTSD patients as identified by ICD9/10 codes (refer to Supplementary information: Appendix A for details) [18, 19].

### Building of RDoC dictionaries

We summarized the current research on PTSD integration into the RDoC framework and built our context-dependent keyword and sentence dictionary from that research and subject matter experts (SME). Artificial intelligence models struggle to classify narratives in niche domains when they have not been trained on them or tailored to the specialized subject matter. We aim to address this problem by including SMEs in dictionary development [20]. Our dictionaries include the following attributes:

#### Negative Valence systems

Research consistently supports the relevance of negative valence systems in PTSD, characterized by fear and anxiety symptoms, and particularly anxious avoidance of trauma-related cues. This anxiety may generalize to neutral cues during flashbacks, and key mechanisms involving the amygdala, prefrontal cortex, and hippocampus are implicated in fear conditioning and extinction [21, 22]. PTSD is associated with overgeneralized fear, impairments in fear extinction, and cue generalization. Dysfunctional amygdala and hypoactivity in the ventromedial prefrontal cortex contribute to heightened fear responses and hindered extinction [23]. Genetic factors, including the BDNF val66met-allele, are linked to impaired fear extinction, impacting treatment response [24].

#### Positive Valence systems

Positive valence systems, focusing on reward learning and valuation, are understudied in PTSD, with anhedonia reflecting emotional numbing and diminished goal-oriented behavior [25]. Reward processing deficits involve dopamine and serotonin systems, influenced by genetic factors [26]. Oxytocin and SSRIs show promise in addressing reward deficits and anhedonia in PTSD treatment [27].

#### Cognitive systems

Cognitive deficits in PTSD affect attention, planning, and memory, with attentional bias towards threat stimuli and memory biases contributing to hyperarousal [28]. Epigenetic modifications and gene polymorphisms, like in the glucocorticoid receptor (GR) gene, are linked to memory deficits in PTSD [29, 30]. Effective treatment may improve cognitive deficits.

#### Arousal and Regulatory systems

Hyperarousal, a core symptom of PTSD, involves heightened nervousness, sleep problems, and increased startle responses, with sympathetic nervous system overdrive contributing [6, 31, 32]. Genetic variations in adrenergic receptors influence emotional memory, and medications like prazosin and propranolol show efficacy in treating PTSD-related hyperarousal [33, 34].

#### Systems for social processes

Social processes, including attachment, communication, and self-perception, are affected in PTSD, particularly in cases of complex PTSD or interpersonal trauma [35]. Concepts like shame, guilt, and paranoid distrust are prevalent in interpersonally traumatized PTSD patients and merit further study [36–38].

#### Sensorimotor systems

Current transdiagnostic research explores sensorimotor abnormalities in children, individuals at risk of psychosis, and first-episode psychosis patients, among others [39–41]. Sensorimotor dysfunction, recognized only in recent years, can be used to enhance early identification and develop effective treatments.

**Fig. 1:**
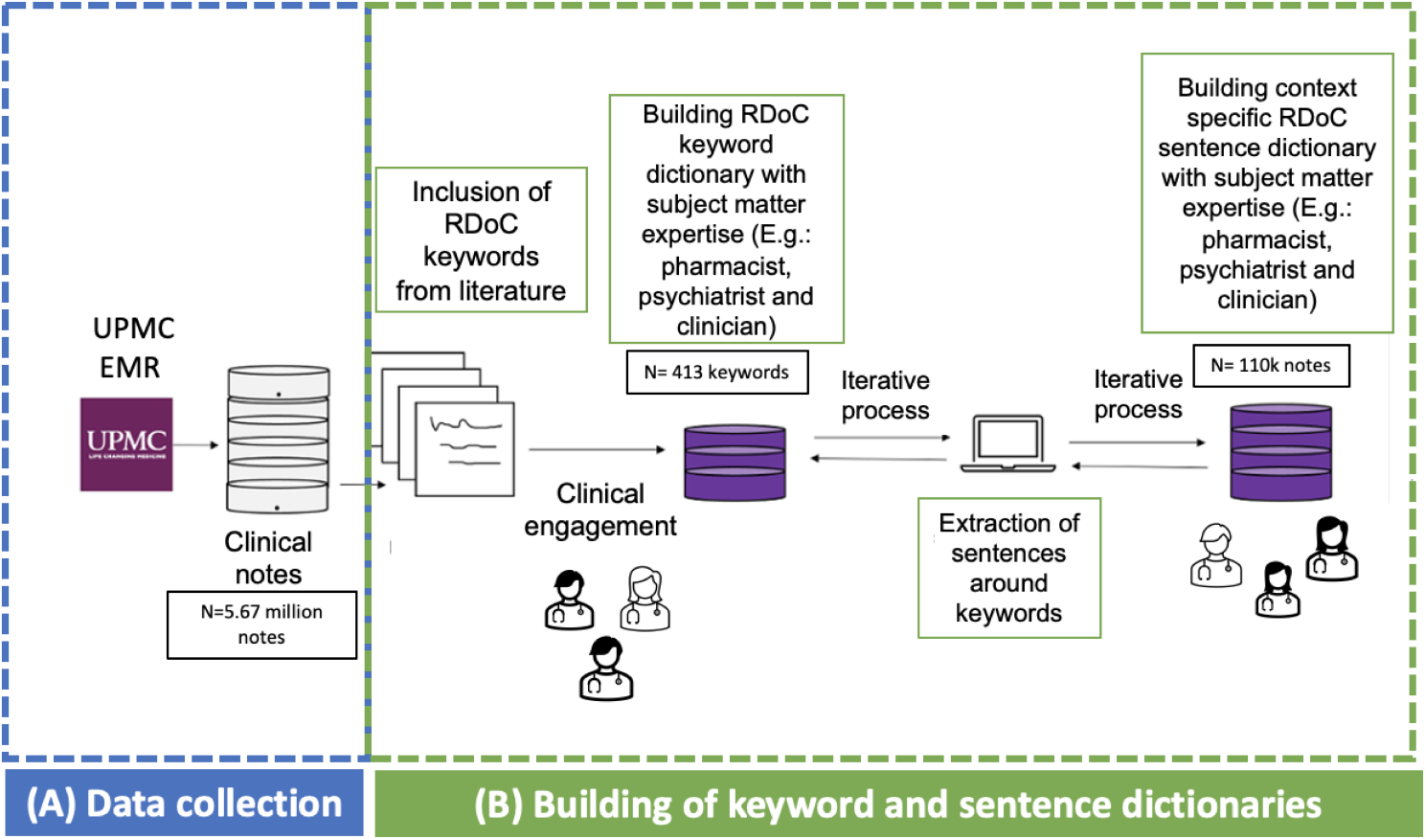
Iterative flow of our sentence dictionary development during ad-hoc collection, clinical engagement, and data integration. The steps of the iterative workflow correspond to. (**A**) Data collection (**B**) Building of keyword and sentence dictionary from literature [43, 44] and SMEs

**Fig. 2:**
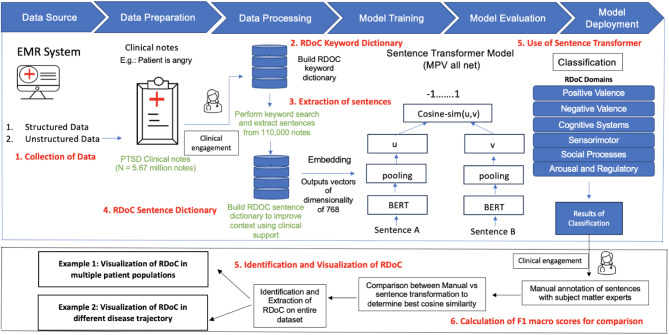
Diagrammatic representation of workflow of our study

RDoC involves studying various domains of human functioning, such as cognition, emotion, and behavior, using a dimensional approach. Since RDoC words often consist of simple English words that carry rich contextual information, traditional deep learning models may struggle to effectively capture their semantic nuances. Sentence transformers, an application of deep learning, excel in understanding the context and semantic similarity of text, making them invaluable for tasks like RDoC classification and analysis studies such as ours. For example, let us consider the RDoC domain of “Negative Valence Systems”, which includes concepts like fear, anxiety, and irritability. While these words are straightforward, their contextual usage and contextual meanings can vary greatly. A sentence transformer can effectively capture these variations and similarities, enabling more accurate analysis and classification of texts related to this domain. Another example is the RDoC domain of “Positive Valence Systems”, which encompasses concepts like reward processing and motivation. Although these words may seem simple on the surface, their contextual usage in different contexts, including clinical notes, requires a much deeper understanding of their semantic nuances. Sentence transformers excel in capturing them. Unlike traditional deep learning models, which may limit classification to a single RDoC domain, sentence transformers offer the flexibility to classify a single sentence into multiple RDoC domains. This capability makes them more versatile and advantageous for our specific task. Utilizing this model may enhance the precision and efficiency of our analysis and multiclass classification tasks within this domain. We utilized a “Human-in-the-Loop” approach, incorporating subject matter expertise to develop these sentence dictionaries [42]. We developed sentence dictionaries to address the limitation of existing keyword dictionaries, which often include common English words lacking context specificity. Through sentence dictionaries, we aim to encompass the entire context in which a word associated with RDoC domain is utilized. Figures 1 and 2 illustrate the iterative flow of sentence dictionary development and the study workflow, respectively. Tables S1 and S2 depict the RDoC keyword and sentence dictionaries. The study aims to identify population and disease-trajectory-specific RDoC domains for early PTSD diagnosis and treatment research.

### Sentence transformer model

Sentence transformers are models used for converting input texts into dense vectors (embeddings) that capture semantic information, allowing for the calculation of semantic similarity between texts using techniques such as cosine similarity. Compared to deep learning models, sentence transformers offer advantages in capturing semantic similarity by representing words with similar meanings closer together in the vector space. Cosine similarity measures the cosine of the angle between two vectors, indicating their similarity or closeness [45]. This approach enables the comparison of semantic similarity by finding the angle between the vectors, where a smaller angle indicates higher similarity, and a larger angle indicates lower similarity. Sentence transformers are important because they can capture the essence of RDoC by considering the context and semantic meaning of words. We used the pre-trained model all-mpnet-base-v2 [46], a transformer-based natural language model to identify the presence of RDoC domains in clinical notes of PTSD patients. The model is based on the MPNet architecture and has the highest performance in generating sentence embeddings according to Sentence-Transformers [20, 46]. We did not perform any additional fine-tuning on our dataset. The model that was provided by Sentence-Transformers was used in its original form, which has an output dimension of 768. Thus, the output of this model for each RDoC in the PTSD dataset is an embedding that has a length of 768.

The RDoC extraction pipeline involves: (1) Collecting and preprocessing of 5.67 million PTSD clinical notes from the UPMC EMR system. (2) Building of a keyword dictionary from literature and SMEs. (3) Conducting keyword searches and extracting sentences from 110,000 clinical notes. (4) Creating a context-specific RDoC sentence dictionary, evaluated by SMEs for categorization and inclusion (Table S3). (5) Utilizing sentence transformers to extract RDoC information by comparing cosine similarity scores between sentences, where Sentence A is the new sentence from a clinical note that is being annotated (E.g.: Patient is overworked, stressed and refused to undergo psychotherapy) and Sentence B is the sentence from the sentence dictionary (E.g: Patient is stressed and refuses psychotherapy). The selection criteria involve identifying the presence of a keyword in both sentences. Once the keyword is identified, the sentence transformer focuses on only those sentences where the keyword is present in sentence B. Subsequently, the transformer assesses the cosine similarity scores between Sentence A and each potential Sentence B until the optimal match is determined. This is a supervised approach. (6) Calculating F1 macro scores to determine the optimal threshold for identifying RDoC categories, comparing manual annotation by SMEs with sentence transformer. Macro F1 scores were used as they provide a balanced evaluation across all RDoC categories, ensuring equal consideration of precision and recall for each category in this multiclass classification problem [7]. Identifying and Visualizing RDoC in two use cases (i) Across multiple patient populations and (ii) Throughout various disease trajectories.

### Statistical analysis

The delineation of RDoC domain categories for data collection underwent an iterative refinement due to the extensive volume of clinical notes, as illustrated in Fig. 2. Initial stages encompassed employing a keyword dictionary, extracting sentences with these keywords, labeling sentences into categories through SMEs, and leveraging sentence transformers for RDoC domain identification along with corresponding keywords. Table S3 shows the count of identified patients, with SMEs reviewing a subset of randomly selected cases (*N* = 8,351 sentences). These procedures were crucial in the labeling process.

SMEs initially developed labels that best defined each domain, but noticed missing keywords, prompting ongoing refinement. Inclusion of these keywords enhanced the dictionary, improving RDoC identification accuracy. In the final iteration, SMEs verified correct assignments. Comparing manual SME annotations with sentence transformer results, Table 1 shows that a 0.3 cosine similarity threshold yielded the best F1 macro scores across all RDoC domains, ensuring an F1 score of at least 80% across all domains (*N* = 8,351 sentences). 

**Table 1:** F1 macro scores to determine the threshold value for identification of RDoC using the sentence transformer model

Threshold Value	0.1	0.2	0.3	0.4	0.5	0.6
Arousal regulation	0.48	0.70	0.90	0.79	0.50	0.47
Cognitive systems	0.17	0.47	0.87	0.89	0.83	0.67
Negative valence	0.17	0.42	0.92	0.95	0.97	0.67
Positive valence	0.17	0.54	0.84	0.81	0.77	0.47
Sensorimotor systems	0.17	0.60	0.83	0.86	0.89	0.96
Social process	0.17	0.66	0.86	0.76	0.50	0.17

## Results

Baseline information, encompassing 12 categories of mental disorders (see Appendix B) [47], age, gender, and the follow up times, is detailed in Table 2. Following the data processing, dictionary development, and application of the sentence transformer (Steps 1–6) as seen in Fig. 2, we identified the Top 10 keywords and their respective counts identified in our dataset (Table S4), and the top keyword along with an example corresponding sentence in our dataset (Table S5).

**Table 2:** Baseline characteristics of the cohort

	Overall
n	38,801
Gender (%)	
Female	24,501 (63.1)
Male	14,294 (36.8)
Unknown	6 ( 0.0)
Age (Mean (SD))	37.81 (16.83)
Race (%)	
Alaska native	7 ( 0.0)
American Indian	179 ( 0.5)
Black	7044 (18.2)
Chinese	26 ( 0.1)
Declined	299 ( 0.8)
Filipino	23 ( 0.1)
Guam/Chamorro	3 ( 0.0)
Hawaiian	9 ( 0.0)
Indian (Asian)	50 ( 0.1)
Japanese	6 ( 0.0)
Korean	16 ( 0.0)
Not specified	514 ( 1.3)
Other Asian	108 ( 0.3)
Other pacific Islander	25 ( 0.1)
Samoan	2 ( 0.0)
Unknown	322 ( 0.8)
Vietnamese	7 ( 0.0)
White	30,161 (77.7)
Follow_up_time (mean (SD))	4.44 (5.71)
Cat1_1Year = 1 (%) [Alcohol-induced mental disorders, Drug-induced mental disorders, Alcohol dependence syndrome, Drug dependence, Nondependent abuse of drugs (not Tobacco use disorder)]	4660 (12.0)
Cat2_1Year = 1 (%) [Schizophrenic disorders, Schizoid personality disorder]	872 ( 2.2)
Cat3_1Year = 1 (%) [Episodic mood disorders, Depressive type psychosis, Dysthymic disorder, Affective personality disorder, Adjustment reaction, Depressive disorder NEC]	14,378 (37.1)
Cat4_1Year = 1 (%) [Delusional disorders, Other nonorganic psychoses (not Depressive type psychosis)]	540 ( 1.4)
Cat5_1Year = 1 (%) [Acute reaction to stress, Anxiety, dissociative and somatoform disorders]	11,560 (29.8)
Cat6_1Year = 1 (%) [Personality disorders (not Affective personality disorder or Schizoid personality disorder)]	861 ( 2.2)
Cat7_1Year = 1 (%) [Sexual and gender identity disorders]	57 ( 0.1)
Cat8_1Year = 1 (%) [Physiological malfunction arising from mental factors, Psychic factor without dis.]	89 ( 0.2)
Cat9_1Year = 1 (%) [Special symptoms or syndromes not elsewhere classified]	940 ( 2.4)
Cat10_1Year = 1 (%) [Dementias, Transient mental disorders due to conditions classified elsewhere, Persistent mental disorders due to conditions classified elsewhere, Specific nonpsychotic mental disorders due to brain damage]	854 ( 2.2)
Cat11_1Year = 1 (%) [Autistic disorder-current, Disturbance of conduct not elsewhere classified, Disturbance of emotions specific to childhood and adolescence, Hyperkinetic syndrome of childhood, Specific delays in development]	2293 ( 5.9)
Cat12_1Year = 1 (%) [Mild intellectual disabilities, Other specified intellectual disabilities, Unspecified intellectual disabilities]	172 ( 0.4)

In mental health research, especially in PTSD, innovative approaches are essential to address gaps in understanding the condition’s complex mechanisms. Traditional methodologies may overlook crucial details in clinical narratives, hindering the identification of nuanced patterns and tailoring interventions. Leveraging the sentence transformer model, we explored PTSD-related information more comprehensively in unstructured data. Emphasizing the crucial role of RDoC identification, two compelling use cases illustrate the potential of this approach:

### Example 1: identification of RDoC domains across different patient populations

**Fig. 3:**
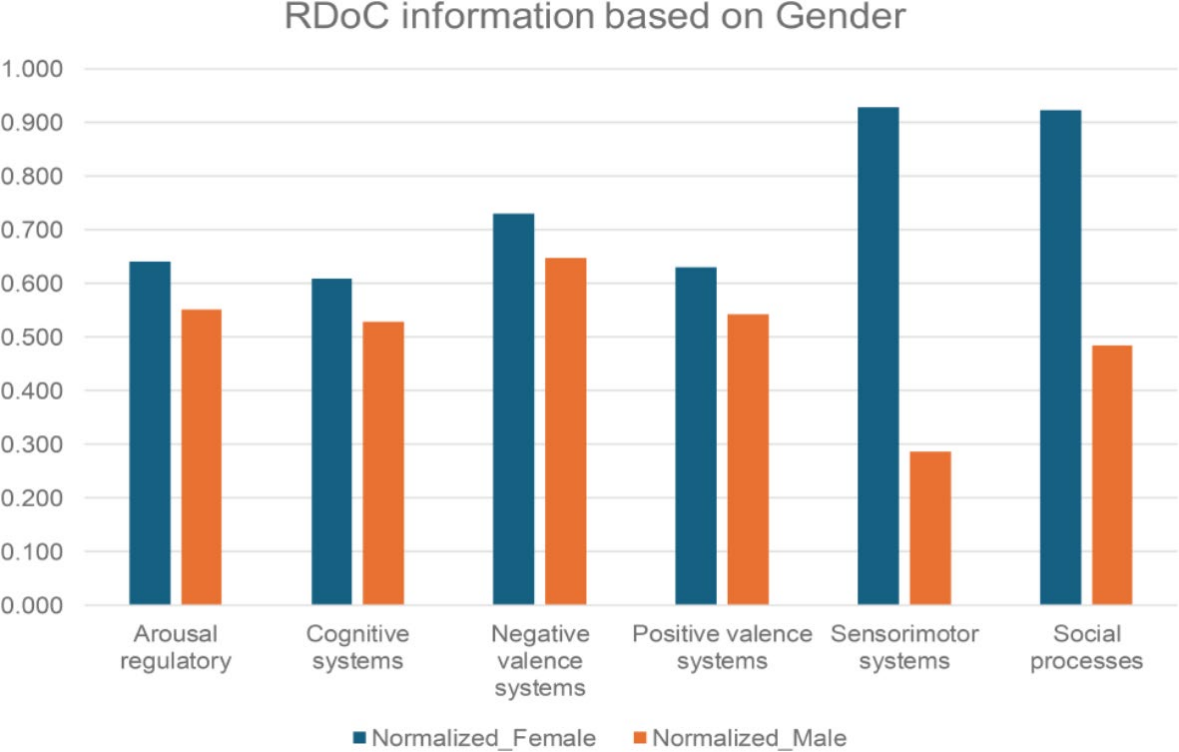
RDoC domains across different patient populations: male and female. *p value = 0.03552 (Wilcoxon signed-rank test)

Our goal is to empower clinicians and researchers by understanding how various RDoC domains manifest in different patient populations, facilitating the development of targeted strategies for unique needs. Analyzing demographic data from structured EMRs, we found females (*N* = 14,862) exhibited a higher prevalence of RDoC information than males (*N* = 7,333) (Fig. 3 and Table S6).

**Fig. 4:**
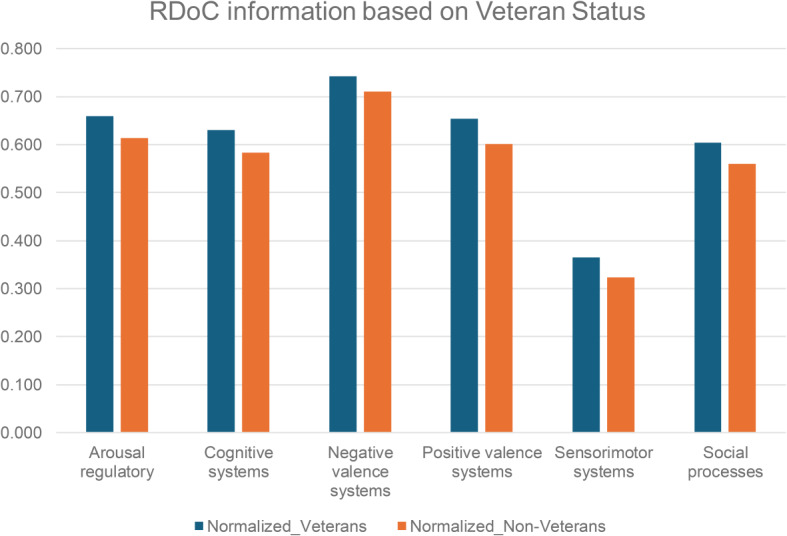
RDoC domains across different patient populations: veterans and non-veterans. *p value = 0.03125 (Wilcoxon signed-rank test)

We analyzed veteran status and psychotherapy details from unstructured EMRs. And explored differences in behaviors, cognitions, and mental health symptoms between veterans (*N* = 4,657) and non-veterans (*N* = 17,114) (Fig. 4 and Table S7), finding the instances of negative valence system constructs, including acute threat (e.g., fear, panic), potential threat (e.g., inhibition, worry), sustained threat (e.g., chronic stress), frustrative non-reward (e.g., reactive aggression), reduced behavioral activation (e.g., anhedonia), and loss (e.g., low well-being), and positive valence system constructs including reward seeking and consummatory behaviors in veterans.

**Fig. 5:**
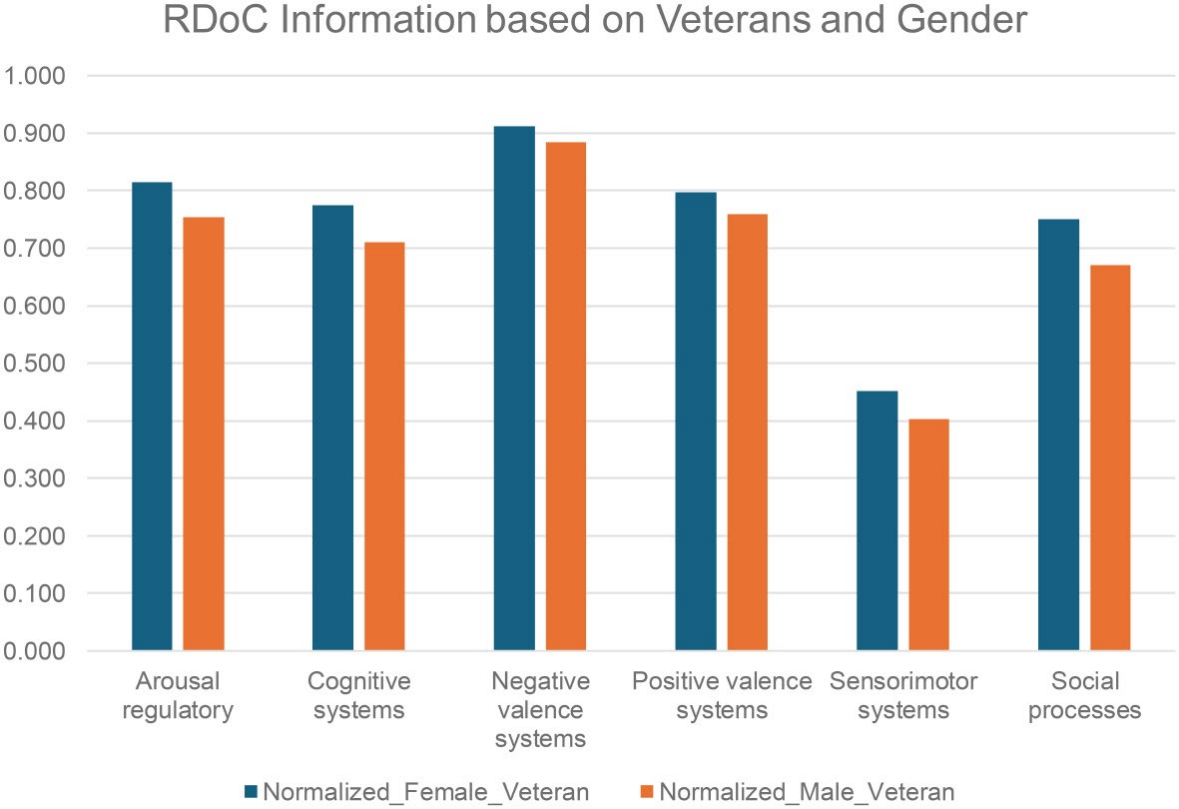
RDoC domains across different patient populations: PTSD patients based on gender and veteran status *p value = 0.03125 (Wilcoxon signed-rank test)

**Fig. 6:**
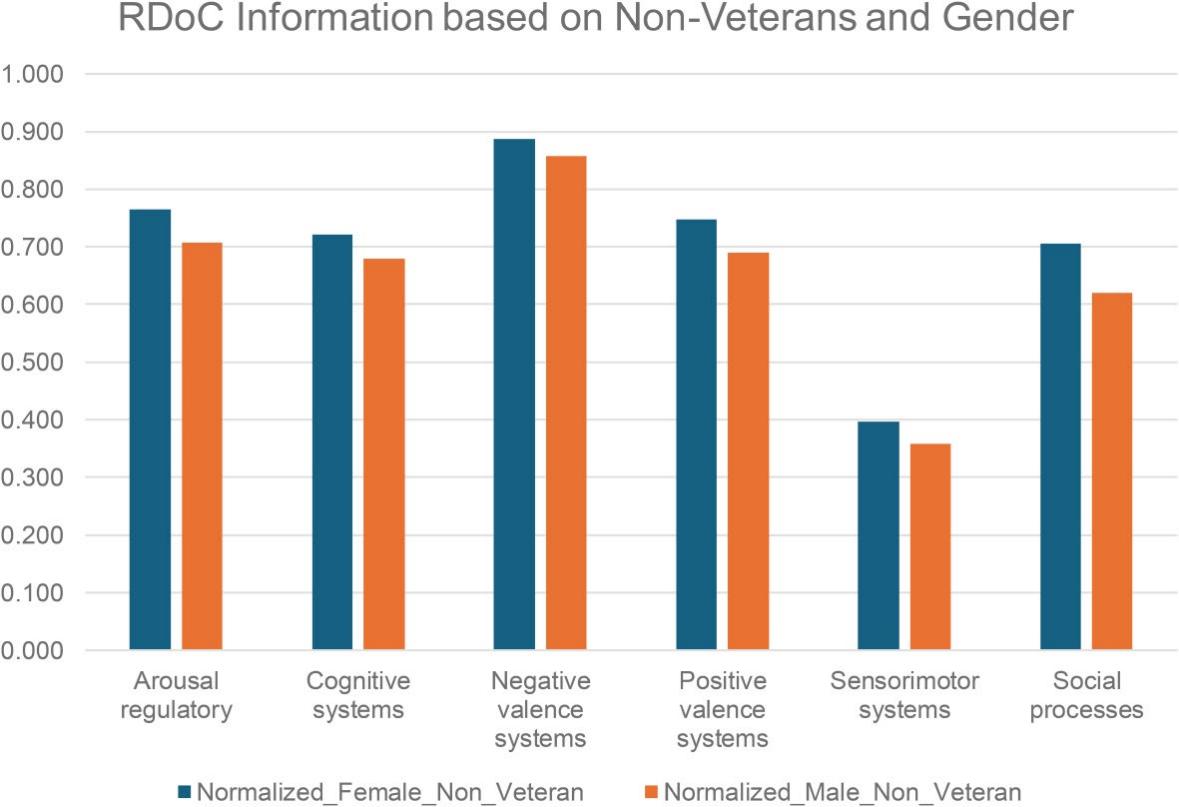
RDoC domains across different patient populations: PTSD patients based on gender and non-veteran status *p value = 0.03125 (Wilcoxon signed-rank test)

We conducted a systematic examination of RDoC domains among PTSD patients, incorporating gender and veteran status (refer to Figs. 5 and 6, and Table S8 and S9), the distinct groups include female veterans (*N* = 2766), male veterans (*N* = 2855), female non-veterans (*N* = 13,775), and male non-veterans (*N* = 5228). The findings revealed fewer occurrences across all RDoC domains in male PTSD patients compared to their female counterparts, a trend persisting even when considering veteran status. This observation highlights potential gender-related differences, extending across both the veteran and non-veteran populations.

**Fig. 7:**
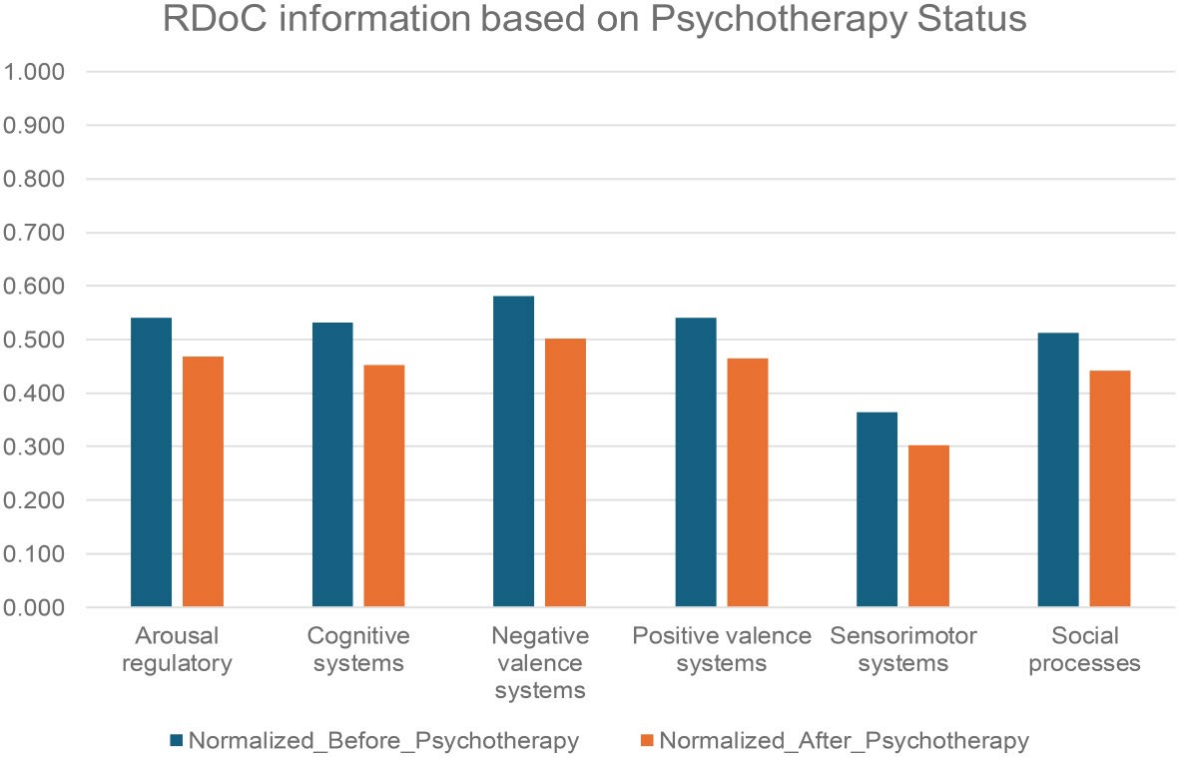
RDoC domains across different patient populations: PTSD patients before psychotherapy and after psychotherapy *p value = 0.03125 (Wilcoxon signed-rank test)

We investigated the differences RDoC domain in PTSD patients before psychotherapy (*N* = 2,262) and after psychotherapy (*N* = 3,189) (Fig. 7 and Table S10). The results revealed a consistent decrease in instances across all RDoC domains in PTSD patients after psychotherapy. This observation highlights the potential positive impact of psychotherapy on alleviating symptoms associated with PTSD. While further investigations are necessary, our findings contribute to advancing PTSD research by shedding a direct positive light on the potential impact of psychotherapy on various RDoC domains.

### Example 2: impact of RDoC on overall disease trajectory

**Fig. 8:**
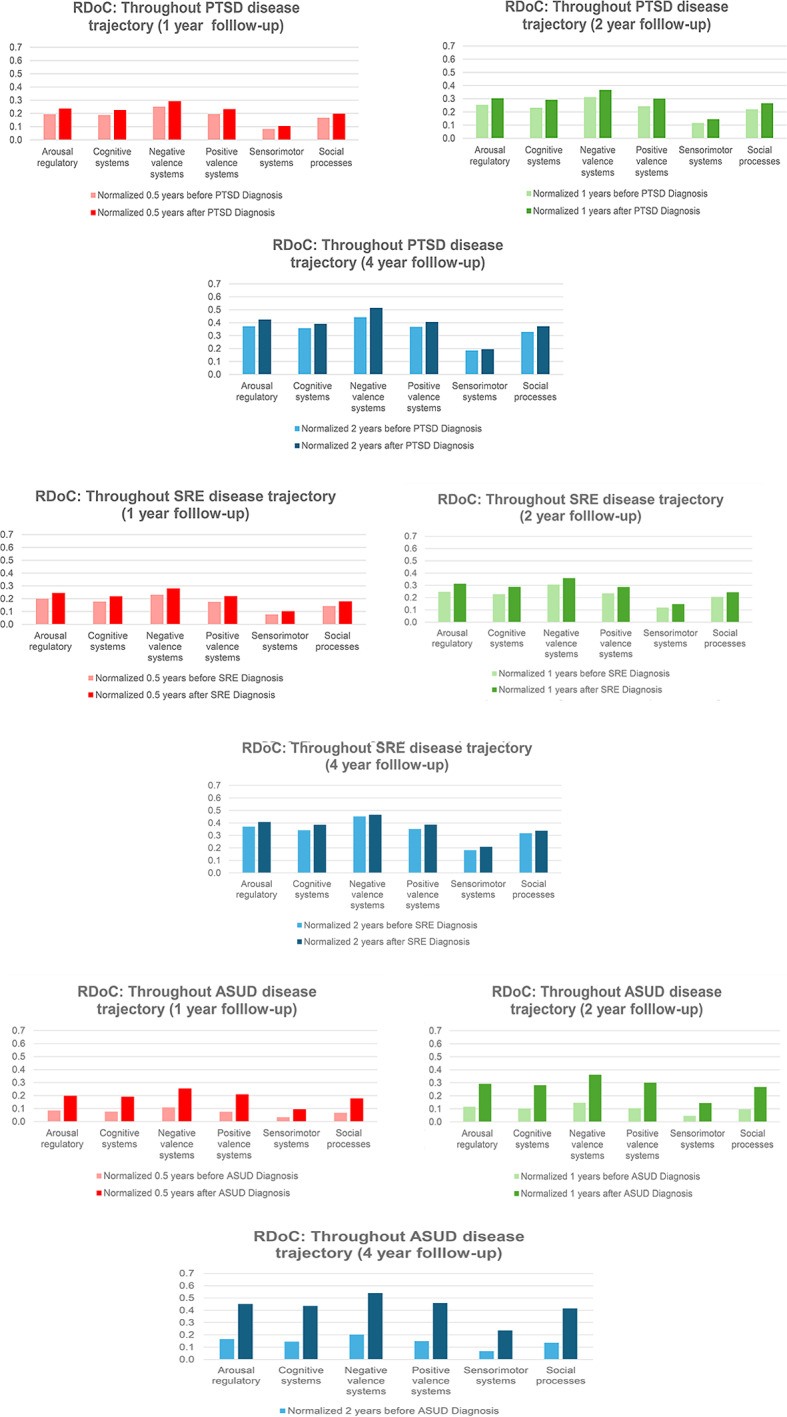
**Overall RDoC changes throughout disease trajectory (PTSD, SRE, ASUD).** *p value = 0.00001475 (Friedman rank sum test). *p value = 0.00001475 (Friedman rank sum test). *p value = 0.00001475 (Friedman rank sum test)

Exploring RDoC domains in the trajectory of PTSD yields valuable insights into the dynamic nature of mental health conditions, allowing researchers to identify critical phases in progression and understand underlying mechanisms. This knowledge is pivotal for developing targeted interventions at specific disease stages, potentially preventing exacerbation, and guiding effective interventions. In our analysis, we identified PTSD patients with first diagnoses of PTSD (*N* = 22,198), suicide-related events (SREs) (*N* = 5,590), and alcohol and substance use disorder (ASUD) (*N* = 13,993) within 1, 2, and 4 years of follow-up (Fig. 8 and Tables S11-S19). RDoC information extracted from unstructured EMR data revealed higher instances of overall RDoC domain changes in SRE-diagnosed patients compared to PTSD-diagnosed patients. Patients with ASUD diagnoses exhibited a substantial increase in RDoC domains, particularly in negative and positive valence systems, over the 4-year follow-up, emphasizing the importance of RDoC identification in studying PTSD sequelae. Further research is needed to comprehend the relationship between negative valence systems and ASUD.

Collectively, these findings highlight the positive impact of identifying RDoC symptoms in different populations and diagnoses. Significantly, these results propose potential advantages in the development and utilization of measures focusing on transdiagnostic factors aligned with the RDoC.

#### World Cloud

**Fig. 9:**
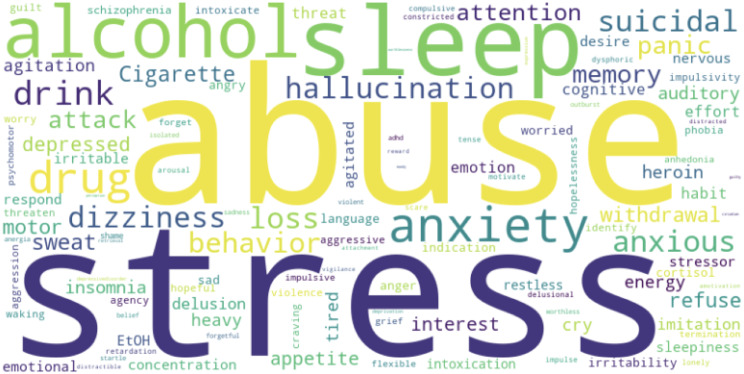
RDoC keywords for PTSD patients

Word clouds visually represent qualitative data, with word size reflecting frequency or significance. Applied in medical literature and beyond, they creatively highlight patterns [48]. Our assessment explored RDoC’s impact on PTSD, using Python (v3.8.8) to generate word clouds. Figure 9 illustrates the prominence of RDoC keywords (e.g., stress, abuse, alcohol, sleep, suicidal, anxiety, hallucination, and attention) using all the clinical notes of the PTSD population, transcending specific domains. Supplementary information details each keyword within its RDoC domain (Figures S1-6).

The word cloud was crafted from the clinical notes of PTSD patients, capturing insights related to RDoC domains. The magnitude of each word is directly linked to the number of instances during the analysis.

## Discussion

Our study delves into PTSD within the RDoC framework, exploring if multimodal patient information aligns with RDoC domains. It uniquely examines functioning RDoC domain alterations in PTSD patients across diverse populations. Utilizing custom dictionaries, RDoC was identified as six domains (Cognitive, Positive Valence, Negative Valence, Social Processes, Sensorimotor, and Arousal/Regulatory Systems). The study’s approach, leveraging existing measures, demonstrates promise in utilizing artificial intelligence tools for context-specific RDoC domain identification in neuropsychiatric research and promotes integration of research discoveries to offer a potentially valuable dimensional perspective on patients diagnosed with PTSD.

In line with our hypotheses, targeted domains increased in severity post-PTSD diagnosis, consistent with findings by Coffey et al. indicating a general shift in symptoms across specific diagnostic categories including substance use, depression, and anxiety [49, 50]. Our approach aligns with RDoC’s goal of uncovering underlying psychopathological mechanisms, departing from traditional categories to consider multimodal information for personalized treatment [51]. Analyzing pre-diagnosis values, we observed a rise in RDoC symptoms in patients diagnosed two years later compared to two years before PTSD diagnosis, over a 4-year follow-up. Lower RDoC domain scores were associated with reduced distress, sadness, aggression, worry, anhedonia, alcohol, and substance cravings. Conceptually grouping these symptoms within functioning domains, we identified associations such as distress and sadness with the arousal and regulatory system, aggression with social processing, anhedonia with cognitive system, worry with negative valence system, and alcohol and substance craving with the positive valence system, assessing overall domain functioning in these patients.

The Cognitive systems domain showed a reduced number of instances in PTSD patients after psychotherapy, aligning with the notion that subjective distress, uncontrollability, and unpredictability are key anxiety components [52]. Veterans, women, and those before psychotherapy exhibited higher severity. Psychotherapy, addressing dysfunctional trauma-related cognitions, led to beneficial reductions in emotional distress, emphasizing the interplay between cognitive and affective systems. Further cognitive-specific therapies may enhance cognitive restructuring [53]. Negative Valence Systems instances peaked in PTSD patients before psychotherapy but decreased after psychotherapy, linked to reduced subjective distress and alcohol cravings [54]. This highlights the RDoC domain’s role in PTSD outcomes. Positive Valence systems showed higher instances in veterans and PTSD patients before psychotherapy, suggesting reward system dysfunction [25]. Trauma-related abnormalities in neural structure contribute to symptoms like anhedonia and reduced motivation [55]. PTSD, more prevalent in women, involves dysregulated neuronal, hormonal, and immune mechanisms, impacting sensory processing and sensorimotor systems [56]. Abnormal sensorimotor occurrences, potentially linked to prepulse inhibition (PPI), were more prominent in females. PPI, a measure of sensorimotor filtering, has been associated with conditions like challenges in suppressing sensory or motor information. A study found that trauma-exposed women with PTSD exhibited deficits in PPI, supporting our empirical findings of sensorimotor abnormalities in individuals with PTSD [57]. Social cognition encompasses processes linking the perception of social information to behavioral responses, including perception, attention, decision-making, memory, and emotion [58]. Social processing deficits such as fear responses associated with trauma may be alleviated by psychotherapy, emphasizing its positive effect. Arousal/Regulatory domain scores decreased in PTSD patients after psychotherapy, highlighting the significance of physiological symptoms. Sleep patterns impact distress and the Arousal/Regulatory domain, emphasizing their role in PTSD development [59]. This aligns with findings from another study [60], suggesting the need for veteran-specific RDoC markers, though further research is warranted.

Additionally, we found lower abnormal instances of RDoC male/ non-veterans/ male non-veterans when compared to females/veterans/female non-veterans. The lower incidence of abnormal instances of RDoC in males, non-veterans, and male non-veterans could be attributed to various factors. It’s possible that the prevalence or severity of the conditions captured by RDoC domains differs between demographic groups. Additionally, differences in healthcare-seeking behavior, access to healthcare services, social and cultural factors, or even genetic predispositions could contribute to these disparities.

Our research is pivotal in advancing PTSD studies by employing natural language processing techniques to extract RDoC information from unstructured EMR data. This approach enhances our understanding of subtle variations in symptomatology, contributing to a more comprehensive view of PTSD. Additionally, our study highlights the importance of personalized and context-specific mental health interventions. Identifying RDoC domains allows tailored interventions for specific patient populations and disease stages, addressing unique challenges. This personalized approach holds the potential to significantly enhance treatment outcomes and patient well-being. Overall, our research not only addresses critical gaps in PTSD research methodology but also informs policy decisions by providing evidence-based insights to improve resource allocation, tailor support, shape comprehensive policies and engage stakeholders, ensuring the translation of RDoC identification benefits into meaningful advancements in mental healthcare.

Our study has notable limitations that warrant consideration. In constructing domains aligned with RDoC, we repurposed words from existing measures due to the absence of validated measures explicitly designed for RDoC domains. While our use of artificial intelligence introduces potential measurement error, the validation by SMEs demonstrated satisfactory results. Data constraints from UPMC, limited to routine medical notes, excluded special mental health reports hindering the incorporation of RDoC measures and further validation. Our focus on five out of the six RDoC domains, particularly in the context of PTSD, raises questions about the applicability of the sixth domain (Sensorimotor Systems). Additionally, the study’s scope is confined to PTSD, and the specific impacts of various treatments or substance use on observed changes remain unclear. Despite these limitations, our study contributes to RDoC’s short term objectives by pioneering measurement tools for its domains, offering valuable insights for future assessments within the framework.

Considering both strengths and limitations, future research should focus on developing valid and reliable measures for RDoC domains, aligning with the RDoC matrix’s comprehensive, multi-unit analysis. Integrating RDoC data into deep-learning models predicting adverse events (e.g. substance use disorder, opioid use disorder, suicide related events) could enhance measure formulation. Expanding the investigation to cover all six RDoC domains is crucial, allowing exploration of associations between specific functioning domains and more pronounced changes with multimodal information. This insight can refine therapeutic change mechanisms, guiding the development of targeted treatments. Future research should extend beyond PTSD, including diverse populations and treatments, enhancing generalizability, and understanding the applicability of RDoC domains across various clinical contexts.

## Conclusion

This paper delves into a crucial aspect of the ever-expanding significance of RDoC as interventions aimed at leveraging multi-modal real-world data continue to progress. We present a systematic approach for obtaining high-quality data from longitudinally collected clinical notes. Our outlined process encompasses data extraction, acquisition, and preparation for analysis, utilizing a transformer-based model that helps identify RDoC in a context-specific manner. Given the escalating importance of RDoC in the field of neuropsychiatric research, our procedure along with the use cases discussed in this paper hold substantial potential for broad applicability across diverse clinical settings and populations.

### Electronic supplementary material

Below is the link to the electronic supplementary material.

Supplementary Material 1

## Data Availability

The data used in this study were from UPMC under a data use agreement. The authors are not permitted to distribute the data to any third party, but researchers may contact UPMC for data access.
