# Supplementary material for: Enhancing post-traumatic stress disorder patient assessment: leveraging natural language processing for research of domain criteria identification using electronic medical records
Source: BMC Med Inform Decis Mak. 2024 Jun 4;24:154. doi: 10.1186/s12911-024-02554-8 (PMC11151516; doi:10.1186/s12911-024-02554-8)

**Supplementary information:**

**Appendix A.** Diagnosis codes

1. PTSD:

309.81, F43.10, F43.11, F43.12

1. ASUD:

291.0, 291.1, 291.2, 291.3, 291.4, 291.5, 291.8, 291.81, 291.82, 291.89, 291.9, 292.0, 292.11, 292.12, 292.2, 292.81, 292.82, 292.83, 292.84, 292.85, 292.89, 292.9, 357.5, 425.5, 535.30, 535.31, 571.0, 571.1, 571.2, 571.3, 648.30, 648.31, 648.32, 648.33, 648.34, 965.00, 965.01, 965.02, 965.09, 968.5, 969.6, E850.0, E854.1, E860.0, E935.0, E938.5,E939.6,V654.2, 303, 303.0, 303.00, 303.01, 303.02, 303.03, 303.9, 303.90, 303.91, 303.92, 303.93, 304, 304.0, 304.00, 304.01, 304.02, 304.03, 304.1, 304.10, 304.11, 304.12, 304.13, 304.2, 304.20, 304.21, 304.22, 304.23, 304.3, 304.30, 304.31, 304.32, 304.33, 304.4, 304.40, 304.41, 304.43, 304.5, 304.50, 304.51, 304.52, 304.6, 304.60, 304.61, 304.62, 304.63, 304.7, 304.70, 304.71, 304.72, 304.73, 304.8, 304.80, 304.81, 304.82, 304.83, 304.9, 304.90, 304.91, 304.92, 304.93, 305, 305.0, 305.00, 305.01, 305.02, 305.03, 305.1, 305.10, 305.12, 305.13, 305.2, 305.20, 305.21, 305.22, 305.23, 305.3, 305.30, 305.31, 305.33, 305.4, 305.40, 305.41, 305.42, 305.43, 305.5, 305.50, 305.51, 305.52, 305.53, 305.6, 305.60, 305.61, 305.62, 305.63, 305.7, 305.70, 305.71, 305.72, 305.73, 305.8, 305.80, 305.81, 305.83, 305.9, 305.90, 305.91, 305.92, 305.93, F10.10, F10.11, F10.120, F10.121, F10.129, F10.14, F10.151, F10.159, F10.180, F10.188, F10.19, F10.20, F10.21, F10.220, F10.221, F10.229, F10.230, F10.231, F10.232, F10.239, F10.24, F10.250, F10.251, F10.259, F10.26, F10.27, F10.280, F10.288, F10.29, F10.920, F10.921, F10.929, F10.94, F10.951, F10.959, F10.96, F10.97, F10.980, F10.982, F10.988, F10.99, F11.10, F11.11, F11.120, F11.121, F11.129, F11.14, F11.159, F11.188, F11.19, F11.20, F11.21, F11.220, F11.221, F11.222, F11.229, F11.23, F11.24, F11.250, F11.259, F11.288, F11.29, F11.90, F11.921, F11.929, F11.93, F11.94, F11.988, F11.99, F12.10, F12.11, F12.121, F12.122, F12.129, F12.150, F12.151, F12.159, F12.180, F12.188, F12.19, F12.20, F12.21, F12.220, F12.23, F12.250, F12.259, F12.288, F12.29, F12.90, F12.920, F12.921, F12.922, F12.929, F12.959, F12.980, F12.988, F12.99, F13.10, F13.11, F13.129, F13.14, F13.180, F13.188, F13.19, F13.20, F13.21, F13.220, F13.221, F13.229, F13.230, F13.231, F13.232, F13.239, F13.24, F13.259, F13.27, F13.280, F13.29, F13.90, F13.920, F13.921, F13.929, F13.930, F13.931, F13.939, F13.94, F13.97, F13.980, F13.99, F14.10, F14.11, F14.120, F14.121, F14.122, F14.129, F14.14, F14.151, F14.159, F14.180, F14.182, F14.188, F14.19, F14.20, F14.21, F14.220, F14.221, F14.222, F14.229, F14.23, F14.24, F14.250, F14.251, F14.259, F14.280, F14.282, F14.288, F14.29, F14.90, F14.920, F14.921, F14.929, F14.94, F14.951, F14.959, F14.980, F14.988, F14.99, F15.10, F15.11, F15.121, F15.129, F15.14, F15.159, F15.180, F15.188, F15.20, F15.21, F15.220, F15.222, F15.229, F15.23, F15.259, F15.29, F15.90, F15.920, F15.921, F15.929, F15.93, F15.94, F15.950, F15.951, F15.959, F15.980, F15.982, F15.988, F15.99, F16.10, F16.11, F16.129, F16.159, F16.20, F16.21, F16.221, F16.24, F16.259, F16.283, F16.90, F16.921, F16.929, F16.950, F16.959, F16.980, F16.983, F16.988, F16.99, F17.200, F17.201, F17.203, F17.208, F17.209, F17.210, F17.211, F17.213, F17.218, F17.219, F17.220, F17.223, F17.228, F17.229, F17.290, F17.298, F17.299, F18.10, F18.11, F18.19, F18.20, F18.24, F18.90, F19.10, F19.11, F19.120, F19.121, F19.129, F19.14, F19.150, F19.159, F19.180, F19.181, F19.188, F19.19, F19.20, F19.21, F19.221, F19.229, F19.230, F19.231, F19.232, F19.239, F19.24, F19.259, F19.280, F19.29, F19.90, F19.920, F19.921, F19.922, F19.929, F19.930, F19.931, F19.939, F19.94, F19.950, F19.951, F19.959, F19.96, F19.980, F19.982, F19.988, F19.99.

1. SRE:

V62.84,R45.851,E950.3,E956,E950.4,E950.0,E958.8,T14.91,E950.9,E958.9,T14.91XA,E950.5,E950.2,E953.0,E958.1,E953.8,E950.1,E950.7,E952.0,E958.0,E957.1,E957.0,E958.5,E952.1,E955.4,T14.91XD,E950.6,E953.9,E955.0,E957.9,E958.7,E958.3,E954,T14.91XS,E951.0,E951.8,E952.8,E953.1,E955.1,E958.6,E958.2,E955.9,E955.2,X83.8XXA,T42.42A,T43.592A,T39.1X2A,X78.8XXA,X78.9XXD,T42.6X2A,X78.9XXA,T43.222A,T50.902A,X83.8XXD,T39.312A,T43.212A,T45.0X2A,X78.1XXA,X78.8XXD,T50.992A,T40.2X2A,T43.292A,T43.012A,T39.012A,T42.8X2A,X78.0XXA,T51.0X2A,T40.5X2A,T40.4X2A,T40.1X2A,T44.7X2A,T38.3X2A,T44.6X2A,T48.1X2A,T46.5X2A,T71.162A,T48.3X2A,T43.022A,T44.3X2A,T50.902D,X79.XXXA,T65.92XA,X78.1XXD,T51.92XA,T42.1X2A,T65.892A,T56.892A,T43.622A,X80.XXXA,T42.4X2D,X78.0XXD,T42.72XA,T43.3X2A,X76.XXXD,T48.4X2A,T51.2X2A,T46.4X2A,T39.1X2D,T40.7X2A,T54.92XA,T40.602A,T45.512A,X76.XXXA,T43.222D,T39.392A,T47.1X2A,T50.902S,X74.9XXD,T39.092A,T38.1X2A,X74.9XXA,T39.312D,T38.892A,T43.612A,X82.8XXA,T42.6X2D,T43.632A,T46.1X2A,T45.0X2D,T50.992D,T54.2X2A,T40.3X2A,T39.012D,T43.4X2A,T58.02XA,T43.592D,X81.0XXA,T43.202A,T43.8X2A,T44.992A,T45.2X2A,T40.1X2D,T41.292A,T50.2X2A,T48.6X2A,T50.7X2A,T49.0X2A,T46.3X2A,T42.0X2A,T36.1X2A,T36.0X2A,X74.9XXS,X72.XXXD,T43.012D,T51.8X2A,T51.0X2D,T54.92XS,T54.3X2A,T65.892D,T65.92XD,T65.222D,T50.3X2A,T48.5X2A,T47.0X2A,T46.6X2A,T65.92XS,T54.1X2A,T52.4X2A,T52.0X2A,T55.1X2A,T59.892A,T42.4X2S,T42.3X2A,T36.3X2A,T37.8X2A,T38.2X2A,T38.3X2D,T40.992A,T40.8X2A,T44.4X2A,T43.692A,T45.2X2D,T44.7X2D,T43.502A,T71.192A,X79.XXXD,X83.2XXA,X83.8XXS,X72.XXXA,X71.9XXA,T48.202A,T40.2X2D,X74.8XXS,T48.3X2D,T39.8X2A,T47.4X2A,T47.6X2A,T50.6X2A,T49.6X2D,T43.3X2D,T50.5X2A,X74.01XA,X73.0XXA,T49.6X2A,X72.XXXS,X78.9XXS,T39.92XA,X80.XXXD,X81.8XXA,T39.4X2A,X77.8XXA,T50.2X2D,T43.622D,T43.292D,T45.4X2A,T46.0X2A,T41.3X2A,T42.5X2A,T42.6X2,T46.7X2A,T46.8X2A,T46.5X2D,T43.1X2A,T43.92XA,T40.5X2D,X71.0XXS,X71.3XXA,X71.8XXA,T43.212D,T46.2X2A,T40.8X2D,T40.602D,T43.022D,T44.1X2A,T46.4X2D,T65.222S,T62.0X2A,T71.162D,T51.1X2A,T51.2X2D,T51.2X2S,T52.8X2A,T51.92XD,T50.8X2A,T56.892D,T58.92XA,T54.3X2S,T54.3X2D,T54.0X2A,T55.0X2A,T36.0X2D,T36.4X2A,T38.5X2A,T36.8X2A,T37.5X2A.

Appendix B. Categories of Co-Morbid Diseases

Category 1 (ICD9: 291* or 292* or 303* or 304* or (305* and not 305.1))

Category 2 (ICD9: 295* or 301.2)

Category 3 (ICD9: 296* or 298.0 or 300.4 or 301.1 or 309* or 311*)

Category 4 (ICD9: 297* or (298* and not 298.0))

Category 5 (ICD9: 308* or (300* and not 300.4))

Category 6 (ICD9: 301* not 301.1 and not 301.2)

Category 7 (ICD9: 302*)

Category 8 (ICD9: 306* or 316*)

Category 9 (ICD9: 307*)

Category 10 (ICD9: 290* or 293* or 294* or 310*)

Category 11 (ICD9: 299* or 312* or 313* or 314* or 315*)

Category 12 (ICD9: 317* or 318* or 319*)

**Appendix: Table A1: Categories of Co-Morbid Diseases**

| **ICD9 Code** | **Disease Name** | **Category** | **ICD9 Code** | **Disease Name** | **Category** |
| --- | --- | --- | --- | --- | --- |
| 291 | Alcohol-induced mental disorders | 1 | 301 (not 301.1 or 301.2) | Personality disorders (not Affective personality disorder or Schizoid personality disorder) | 6 |
| 292 | Drug-induced mental disorders | 1 | 302 | Sexual and gender identity disorders | 7 |
| 303 | Alcohol dependence syndrome | 1 | 306 | Physiological malfunction arising from mental factors | 8 |
| 304 | Drug dependence | 1 | 316 | Psychic factor w oth dis. | 8 |
| 305 | Nondependent abuse of drugs | 1 | 307 | Special symptoms or syndromes not elsewhere classified | 9 |
| (not 305.1) | (not Tobacco use disorder) |  |  |  |  |
| 295 | Schizophrenic disorders | 2 | 290 | Dementias | 10 |
| 301.2 | Schizoid personality disorder | 2 | 293 | Transient mental disorders due to conditions classified elsewhere | 10 |
| 296 | Episodic mood disorders | 3 | 294 | Persistent mental disorders due to conditions classified elsewhere | 10 |
| 298 | Depressive type psychosis | 3 | 310 | Specific nonpsychotic mental disorders due to brain damage | 10 |
| 300.4 | Dysthymic disorder | 3 | 299 | Autistic disorder-current | 11 |
| 301.1 | Affective personality disorder | 3 | 312 | Disturbance of conduct not elsewhere classified | 11 |
| 309 | Adjustment reaction | 3 | 313 | Disturbance of emotions specific to childhood and adolescence | 11 |
| 311 | Depressive disorder NEC | 3 | 314 | Hyperkinetic syndrome of childhood | 11 |
| 297 | Delusional disorders | 4 | 315 | Specific delays in development | 11 |
| 298 | Other nonorganic psychoses | 4 | 317 | Mild intellectual disabilities | 12 |
| (but not 2980) | (not Depressive type psychosis) |  |  |  |  |
| 308 | Acute reaction to stress | 5 | 318 | Other specified intellectual disabilities | 12 |
| 300 | Anxiety, dissociative and somatoform disorders | 5 | 319 | Unspecified intellectual disabilities | 12 |
| (but not 300.4) | (not Dysthymic disorder) |  |  |  |  |

**Table S1. RDoC Domain-Specific Keyword Dictionary**

| **Arousal Regulatory System** | **Negative Valence System** | **Positive Valence System** |
| --- | --- | --- |
| Sadness, sarcasm, satiation, self-assessment, self-consciousness, self-knowledge, self-monitoring, self-perception, self-referential, self-understanding, semantic memory, sensation, sensory arousal threshold, sensory reactivity, separation distress, shame, situation, sleep, sleepiness, sleep-wake, smell, social anhedonia, social approach, social communication, social motivation, social responsiveness, somatosensory, spatial attention, speech, startle, stereotypic, stimulus detection, story learning, strain, stranger, Stroop, SUD, suicidal, suppression, sustained attention, sustained threat, switching, therapy, thought, threat, total sleep time, uncertainty, understanding, updating, valuation, visual hallucination, visual acuity, vocalization, wakefulness, wake time, waking, withdrawal, worried, worry. | Aberrant, abuse, anger, angry, anxiety disorder, anxious, aphasic, appetite, ashamed, attack, attentive, auditory hallucination, avoidant, behavior, belief, blackout, calm, chest pain, compare, compliance, compulsive, concentration, constricted, continuity, conversation, conviction, current episode, deficit, delusional, dependence, depressed, depressive, depressive disorder, deprivation, diaphoretic, diarrhea, difficulty, disinhibit, disorganization, disorganized behavior, distracted, distractible, dysphoric, dysthymic, elicit, empathic, engage, EtOH, fatigue, fearful, feelings, flight, forget, forgetful, frighten, gamble, grandiose, grandiosity, guilt, hallucinate, heavy, helpless, heroin, high-risk, idea, inappropriate, incoherent, induce, induce mood, injurious, insertion, insomnia, intoxicate, intoxication, irritable, isolated, melatonin, memory, misunderstand, money, moody, nervous, night, OSA, outburst, palpitation, panic, paranoid, perception, pessimistic, phobia, posttraumatic, prominent, psychotic disorder, remission, restoration, schizophrenia, tearful, tense, these problems, threaten, trauma, ultimately, understand, vague, vigilance, violence, weight gain, worsen, worth, worthless, worthlessness. | Abnormality, agitation, alcohol abuse, alcohol withdrawal, alertness, cigarette, control, cortisol, disorganize, dizziness, emotional, executive function, expressive, fast, financial, guilty, hallucination, hoard, hopeful, hopelessness, identify, illusion, impairment, impulse, impulse control, impulsive behavior, intake, interest, irritability, lonely, lose, meaningless, misinterpret, motivate, motor activity, notice, ongoing, optimistic, panic disorder, paranoia, posttraumatic stress, psychotic, psychotic feature, quantity, reactivity, recall, refuse, repetitive, respond, restless, sad, scare, sensitivity, sensory deficit, sexual, sexual abuse, sleep study, sleepiness, stability, startle, stereotype, strategy, stress, stressor, sweat, tachycardia, thought broadcasting, tingle, tired, tremulous, user, violent, voluntary, waking, withdrawn. |
| **Cognitive System** | **Sensorimotor System** | **Social Processing System** |
| ADHD, affiliation, aggression, agitation, alert, amotivation, analgesia, anergia, anhedonia, animacy, anticipation, anxiety, approach, arousal, assaultive, attachment, attention, attentional, auditory, avoidance, barbiturate, behavioral activation, bereavement, biological motion, brief, capacity, capture, categorization, childhood trauma, circadian, cognition, cognitive, competition, complex span, compulsive behavior, consolidation, craving, cry, declarative, decreased appetitive behavior, decreased libido, delay, delayed reward, delusion, desire, detection, devaluation, disability, discourse, discrimination, distractibility, distraction, effect, emotion, empathy, employment, energy, episodic memory, facial, familiarity, fear, flexible, freezing, frustration, frustrative, gain, generalized reward, gestural expression, gesture comprehension, goal, grief, head turning, heart rate variability, hypervigilant, imitation, impulsive, impulsivity, ingestion, language, learning, limited capacity, listening, listening time, list learning, perseverative, threat. | Action, adaptation, apathy, calculation, dynamic, execution, habit, hyposensitivity, inhibition, initiation, motor, motor control, motor action, move, perseveration, planning, psychomotor, reaction time, response inhibition, retardation, selection, sensorimotor, sensory, temporal, termination. | Conflict, local, loss, loss drive, maintenance, manipulation, masking, mental state, metaphor, mimicry, monitoring, morbid, motivated behavior, movement, multimodal, multi-stability, naming, nausea, neurobehavioral, noise, non-facial, nonlocal, non-reward, off-task, olfactory, others, ownership, perceptual, performance, phobic, physical aggression, possibly, postural expression, potential threat, priming, probabilistic, probability, punishment, reading, recognition, reinforcement learning, relational aggression, relaxed, repetitive behavior, representation, response, retrieval, reward, rhythm, risk, risky family, routine, rumination, sleep latency, social support. |

**Table S2. Representative sentences of RDoC Sentence Dictionary**

| **RDoC Domains** | **Examples of Sentences in Dictionary** |
| --- | --- |
| **Arousal Regulatory System** | **Examples:**  Suicidal: Endorsed suicidal thoughts with a plan to overdose on medications  Withdrawal: Having difficulties with withdrawal symptoms |
| **Cognitive System** | **Examples:**  Threat: Patient made suicide threat  Hallucinations: Pregnant, admitted to hospital for auditory hallucinations |
| **Negative Valence System** | **Examples:**  Insomnia: Noted chronic issues with insomnia patient self-medicates with daily heavy marijuana use  Aggressive: Admitted for aggressive behavior |
| **Positive Valence System** | **Examples:**  Motivated: Motivated to get better for children  Identify: The patient identify alcohol use as a problem |
| **Sensorimotor System** | **Examples:**  Retardation: 45 y/o male with pmhx of retardation and tobacco abuse Psychomotor: Psychomotor slowing or agitation |
| **Social Processing System** | **Examples:**  Loss: Loss of a child  Panic: Missing work due to panic attack |

**Table S3. Number of patients identified by the Sentence Transformer and number of randomly selected cases to review and manually annotated by subject matter experts**

| **RDoC** | **No. of Patient cases** | **Randomly selected cases to review and manually annotated by Subject matter experts** |
| --- | --- | --- |
| Arousal regulation | 18724 | 443 |
| Cognitive systems | 17740 | 2165 |
| Negative valence | 21829 | 2629 |
| Positive valence | 18360 | 2797 |
| Sensorimotor systems | 9770 | 74 |
| Social process | 17014 | 243 |

**Table S4. Top 10 keywords in each RDoC domain.**

**
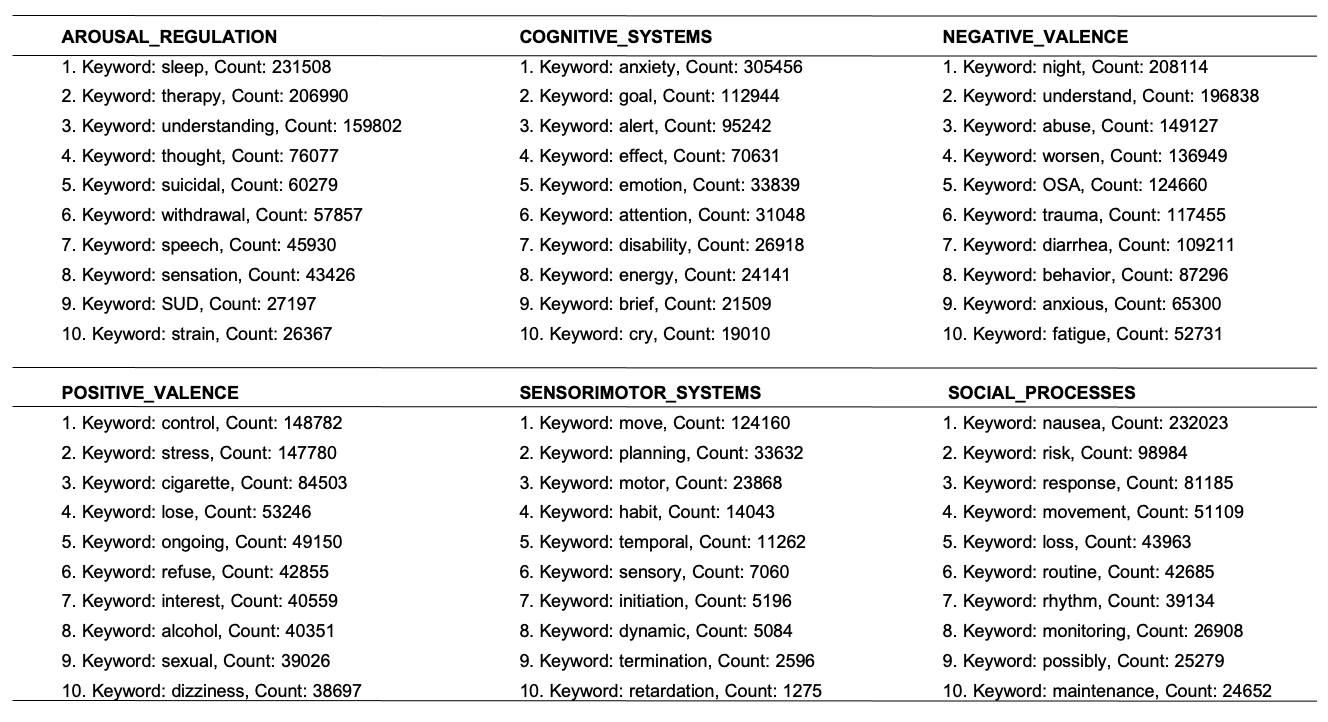
**

**Table S5. Top keyword and sentence in each RDoC domain:**

| **Domain** | **Keyword** | **Sentence** |
| --- | --- | --- |
| Arousal regulation | Sleep | pain woke from sleep, take valium |
| Cognitive systems | Anxiety | psychiatric history: history of anxiety and depression |
| Negative valence | Night | reports nightmares/flashbacks, trial of prazosin to target nightmares |
| Positive valence | Control | eventually his symptoms were controlled |
| Sensorimotor systems | Move | worsens with movement |
| Social process | Nausea | missed appointment due to nausea/vomiting |

**Table S6. RDoC domains across different patient populations: Male and Female**

| **Domain** | **Male** | **Female** | **Normalized Male** | **Normalized Female** |
| --- | --- | --- | --- | --- |
| **Arousal regulation** | 4055 | 9533 | 0.552 | 0.641 |
| **Cognitive systems** | 3875 | 9046 | 0.528 | 0.609 |
| **Negative valence** | 4746 | 10862 | 0.647 | 0.731 |
| **Positive valence** | 3983 | 9360 | 0.543 | 0.630 |
| **Sensorimotor systems** | 2101 | 5125 | 0.287 | 0.928 |
| **Social process** | 3554 | 8846 | 0.485 | 0.923 |

**Table S7. RDoC domains across different patient populations: Veterans and Non-Veterans**

| **Domain** | **Veterans** | **Non-Veterans** | **Normalized Veterans** | **Normalized Non-Veterans** |
| --- | --- | --- | --- | --- |
| **Arousal regulation** | 3073 | 10505 | 0.660 | 0.614 |
| **Cognitive systems** | 2933 | 9988 | 0.630 | 0.584 |
| **Negative valence** | 3460 | 12148 | 0.743 | 0.710 |
| **Positive valence** | 3047 | 10296 | 0.654 | 0.602 |
| **Sensorimotor systems** | 1701 | 5525 | 0.365 | 0.323 |
| **Social process** | 2815 | 9585 | 0.604 | 0.560 |

**Table S8. RDoC domains across different patient populations: PTSD patients based on gender and veteran status**

| **Domain** | **Female Veterans** | **Male**  **Veterans** | **Normalized Female Veterans** | **Normalized Male**  **Veterans** |
| --- | --- | --- | --- | --- |
| **Arousal regulation** | 2255 | 2154 | 0.815 | 0.754 |
| **Cognitive systems** | 2142 | 2027 | 0.774 | 0.710 |
| **Negative valence** | 2520 | 2524 | 0.911 | 0.884 |
| **Positive valence** | 2207 | 2168 | 0.798 | 0.759 |
| **Sensorimotor systems** | 1249 | 1153 | 0.452 | 0.404 |
| **Social process** | 2077 | 1912 | 0.751 | 0.670 |

**Table S9. RDoC domains across different patient populations: PTSD patients based on gender and non-veteran status**

| **Domain** | **Female Non-Veterans** | **Male**  **Non-Veterans** | **Normalized Female Non-Veterans** | **Normalized Male**  **Non-Veterans** |
| --- | --- | --- | --- | --- |
| **Arousal regulation** | 10532 | 3699 | 0.765 | 0.708 |
| **Cognitive systems** | 9945 | 3548 | 0.722 | 0.679 |
| **Negative valence** | 12209 | 4485 | 0.886 | 0.858 |
| **Positive valence** | 10303 | 3602 | 0.748 | 0.689 |
| **Sensorimotor systems** | 5456 | 1876 | 0.396 | 0.359 |
| **Social process** | 9712 | 3243 | 0.705 | 0.620 |

**Table S10. RDoC domains across different patient populations: PTSD patients before psychotherapy and after psychotherapy**

| **Domain** | **PTSD patients before psychotherapy** | **PTSD patients after psychotherapy** | **Normalized PTSD patients before psychotherapy** | **Normalized PTSD patients after psychotherapy** |
| --- | --- | --- | --- | --- |
| **Arousal regulation** | 1223 | 1493 | 0.541 | 0.468 |
| **Cognitive systems** | 1204 | 1441 | 0.532 | 0.452 |
| **Negative valence** | 1314 | 11602 | 0.581 | 0.502 |
| **Positive valence** | 1224 | 1482 | 0.541 | 0.465 |
| **Sensorimotor systems** | 825 | 964 | 0.365 | 0.302 |
| **Social process** | 1157 | 1407 | 0.511 | 0.441 |

**Table S11. RDoC domains across PTSD disease trajectory with a 4-year follow-up period**

| **Domain** | **2 years before PTSD Diagnosis** | **2 years after PTSD Diagnosis** | **Normalized 2 years before PTSD Diagnosis** | **Normalized 2 years after PTSD Diagnosis** |
| --- | --- | --- | --- | --- |
| **Arousal regulation** | 9410 | 8255 | 0.372 | 0.424 |
| **Cognitive systems** | 8693 | 7947 | 0.358 | 0.392 |
| **Negative valence** | 11413 | 9820 | 0.442 | 0.514 |
| **Positive valence** | 9033 | 8167 | 0.368 | 0.407 |
| **Sensorimotor systems** | 4345 | 4117 | 0.185 | 0.196 |
| **Social process** | 8254 | 7310 | 0.329 | 0.372 |

**Table S12. RDoC domains across PTSD disease trajectory with a 2-year follow-up period**

| **Domain** | **1 years before PTSD Diagnosis** | **1 years after PTSD Diagnosis** | **Normalized 1 years before PTSD Diagnosis** | **Normalized 1 years after PTSD Diagnosis** |
| --- | --- | --- | --- | --- |
| **Arousal regulation** | 5634 | 6738 | 0.254 | 0.304 |
| **Cognitive systems** | 5158 | 6501 | 0.232 | 0.293 |
| **Negative valence** | 6940 | 8152 | 0.313 | 0.367 |
| **Positive valence** | 5399 | 6670 | 0.243 | 0.300 |
| **Sensorimotor systems** | 2573 | 3212 | 0.116 | 0.145 |
| **Social process** | 4890 | 5892 | 0.220 | 0.265 |

**Table S13. RDoC domains across PTSD disease trajectory with a 1-year follow-up period**

| **Domain** | **0.5 years before PTSD Diagnosis** | **0.5 years after PTSD Diagnosis** | **Normalized 0.5 years before**  **PTSD Diagnosis** | **Normalized 0.5 years after PTSD Diagnosis** |
| --- | --- | --- | --- | --- |
| **Arousal regulation** | 3771 | 5236 | 0.194 | 0.236 |
| **Cognitive systems** | 3441 | 5013 | 0.189 | 0.226 |
| **Negative valence** | 4730 | 6509 | 0.250 | 0.293 |
| **Positive valence** | 3623 | 5178 | 0.196 | 0.233 |
| **Sensorimotor systems** | 1580 | 2329 | 0.083 | 0.105 |
| **Social process** | 3181 | 4406 | 0.167 | 0.198 |

**Table S14. RDoC domains across SRE disease trajectory with a 4-year follow-up period**

| **Domain** | **2 years before SRE Diagnosis** | **2 years after SRE Diagnosis** | **Normalized 2 years before SRE Diagnosis** | **Normalized 2 years after SRE Diagnosis** |
| --- | --- | --- | --- | --- |
| **Arousal regulation** | 2070 | 2278 | 0.370 | 0.408 |
| **Cognitive systems** | 1906 | 2150 | 0.341 | 0.385 |
| **Negative valence** | 2525 | 2601 | 0.452 | 0.465 |
| **Positive valence** | 1965 | 2155 | 0.352 | 0.386 |
| **Sensorimotor systems** | 1014 | 1163 | 0.181 | 0.208 |
| **Social process** | 1773 | 1887 | 0.317 | 0.338 |

**Table S15. RDoC domains across SRE disease trajectory with a 2-year follow-up period**

| **Domain** | **1 years before SRE Diagnosis** | **1 years after SRE Diagnosis** | **Normalized 1 years before SRE Diagnosis** | **Normalized 1 years after SRE Diagnosis** |
| --- | --- | --- | --- | --- |
| **Arousal regulation** | 1388 | 1750 | 0.248 | 0.313 |
| **Cognitive systems** | 1275 | 1608 | 0.228 | 0.288 |
| **Negative valence** | 1712 | 2004 | 0.306 | 0.358 |
| **Positive valence** | 1313 | 1600 | 0.235 | 0.286 |
| **Sensorimotor systems** | 666 | 825 | 0.119 | 0.148 |
| **Social process** | 1151 | 1366 | 0.206 | 0.244 |

**Table S16. RDoC domains across SRE disease trajectory with a 1-year follow-up period**

| **Domain** | **0.5 years before SRE Diagnosis** | **0.5 years after SRE Diagnosis** | **Normalized 0.5 years before SRE Diagnosis** | **Normalized 0.5 years after SRE Diagnosis** |
| --- | --- | --- | --- | --- |
| **Arousal regulation** | 971 | 1368 | 0.200 | 0.245 |
| **Cognitive systems** | 880 | 1227 | 0.178 | 0.219 |
| **Negative valence** | 1209 | 1563 | 0.231 | 0.280 |
| **Positive valence** | 904 | 1229 | 0.175 | 0.220 |
| **Sensorimotor systems** | 434 | 571 | 0.077 | 0.102 |
| **Social process** | 779 | 996 | 0.142 | 0.178 |

**Table S17. RDoC domains across ASUD disease trajectory with a 4-year follow-up period**

| **Domain** | **2 years before ASUD Diagnosis** | **2 years after ASUD Diagnosis** | **Normalized 2 years before ASUD Diagnosis** | **Normalized 2 years after ASUD Diagnosis** |
| --- | --- | --- | --- | --- |
| **Arousal regulation** | 2313 | 6312 | 0.165 | 0.451 |
| **Cognitive systems** | 2028 | 6095 | 0.145 | 0.436 |
| **Negative valence** | 2835 | 7561 | 0.203 | 0.540 |
| **Positive valence** | 2088 | 6428 | 0.149 | 0.459 |
| **Sensorimotor systems** | 950 | 3304 | 0.068 | 0.236 |
| **Social process** | 1895 | 5809 | 0.135 | 0.415 |

**Table S18. RDoC domains across ASUD disease trajectory with a 2-year follow-up period**

| **Domain** | **1 years before ASUD Diagnosis** | **1 years after ASUD Diagnosis** | **Normalized 1 years before ASUD Diagnosis** | **Normalized 1 years after ASUD Diagnosis** |
| --- | --- | --- | --- | --- |
| **Arousal regulation** | 1621 | 4089 | 0.116 | 0.292 |
| **Cognitive systems** | 1447 | 3945 | 0.103 | 0.282 |
| **Negative valence** | 2049 | 5063 | 0.146 | 0.362 |
| **Positive valence** | 1468 | 4200 | 0.105 | 0.300 |
| **Sensorimotor systems** | 651 | 2031 | 0.047 | 0.145 |
| **Social process** | 1340 | 3743 | 0.096 | 0.267 |

**Table S19. RDoC domains across ASUD disease trajectory with a 1 year follow up period**

| **Domain** | **0.5 years before ASUD Diagnosis** | **0.5 years after ASUD Diagnosis** | **Normalized 0.5 years before ASUD Diagnosis** | **Normalized 0.5 years after ASUD Diagnosis** |
| --- | --- | --- | --- | --- |
| **Arousal regulation** | 1192 | 2781 | 0.085 | 0.199 |
| **Cognitive systems** | 1068 | 2679 | 0.076 | 0.191 |
| **Negative valence** | 1525 | 3570 | 0.109 | 0.255 |
| **Positive valence** | 1061 | 2927 | 0.076 | 0.209 |
| **Sensorimotor systems** | 469 | 1339 | 0.034 | 0.096 |
| **Social process** | 951 | 2498 | 0.068 | 0.179 |

**Figure S1. RDoC Keywords based on Domain Types: Arousal Regulation**

**
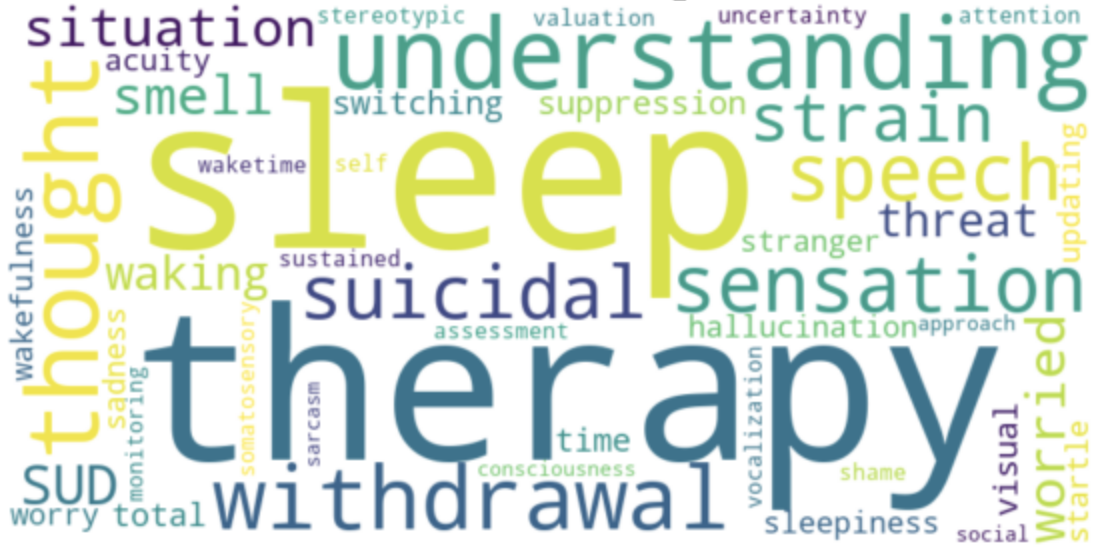
**

**Figure S2. RDoC Keywords based on Domain Types: Cognitive System**

**
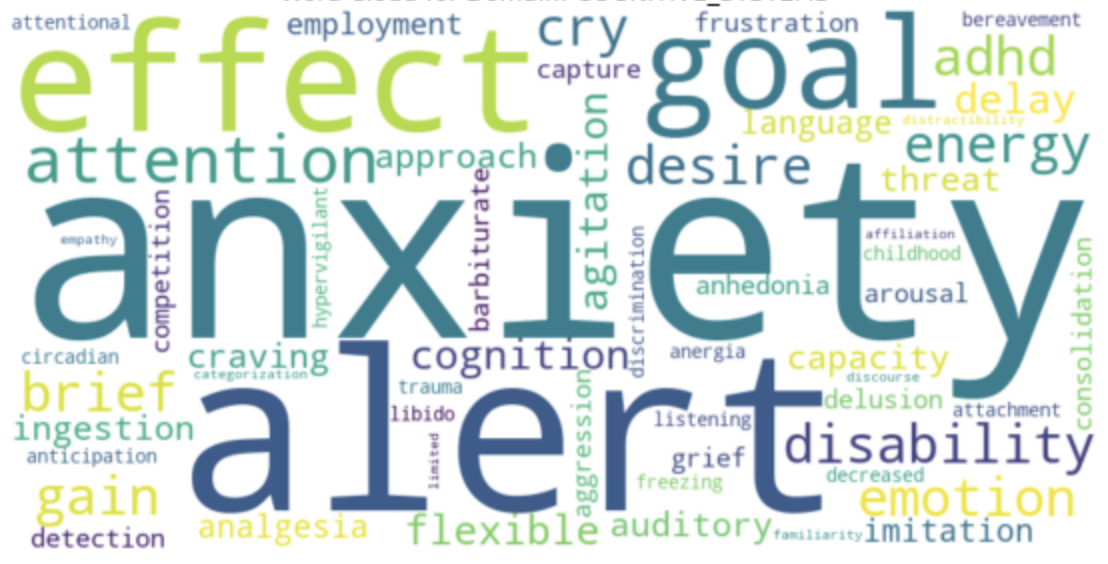
**

**Figure S3. RDoC Keywords based on Domain Types: Negative Valence System**

**
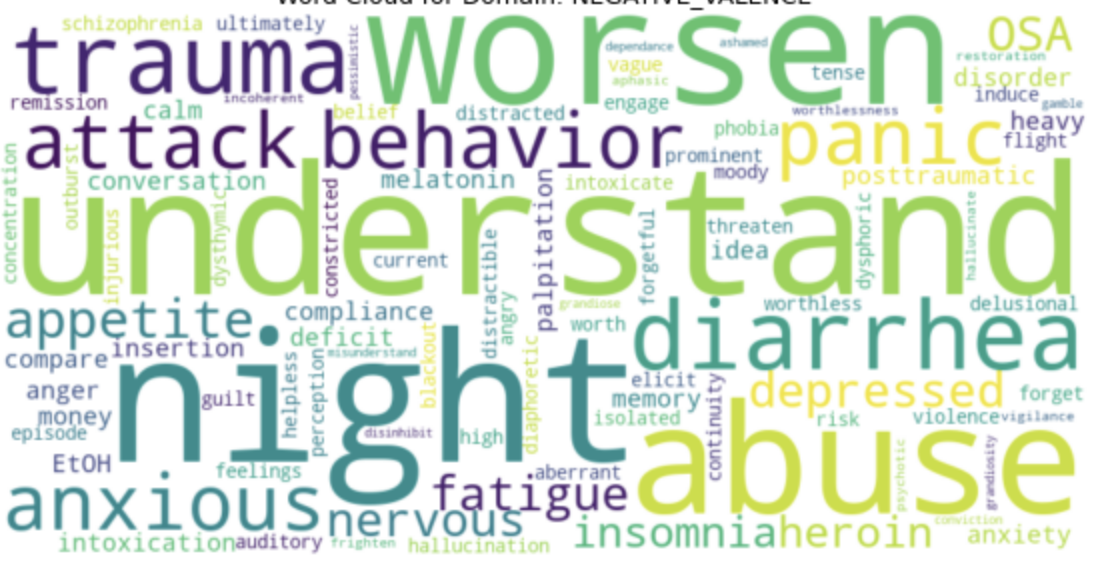
**

**Figure S4. RDoC Keywords based on Domain Types: Positive Valence System**

**
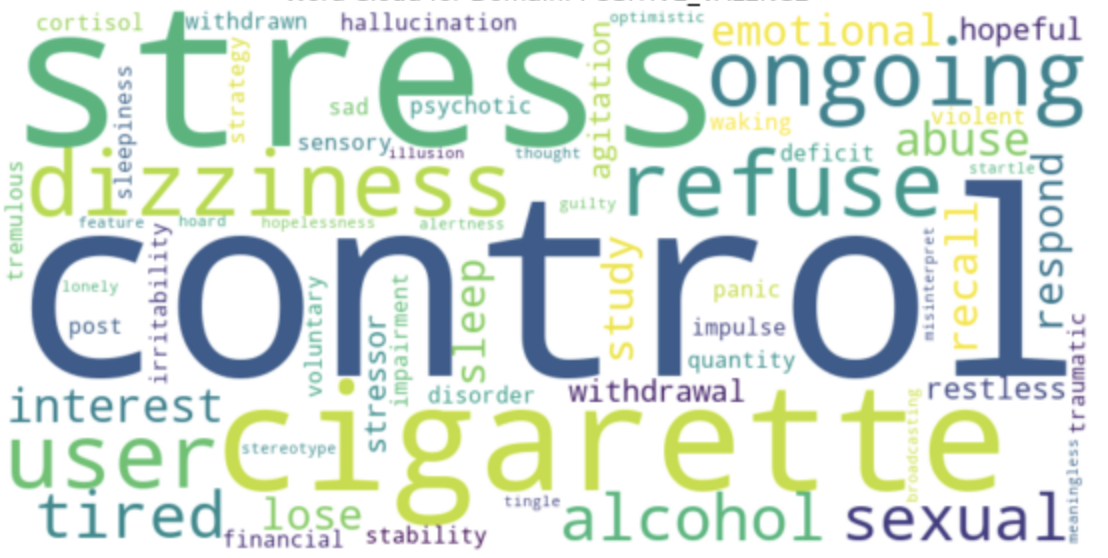
**

**Figure S5. RDoC Keywords based on Domain Types: Sensorimotor Systems**

**
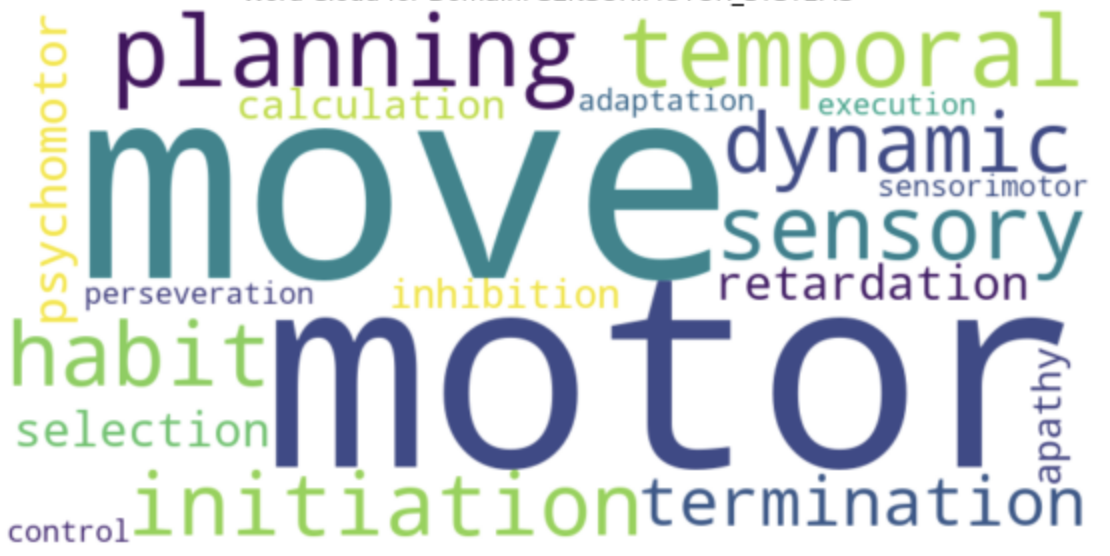
**

**Figure S6. RDoC Keywords based on Domain Types: Social Process**


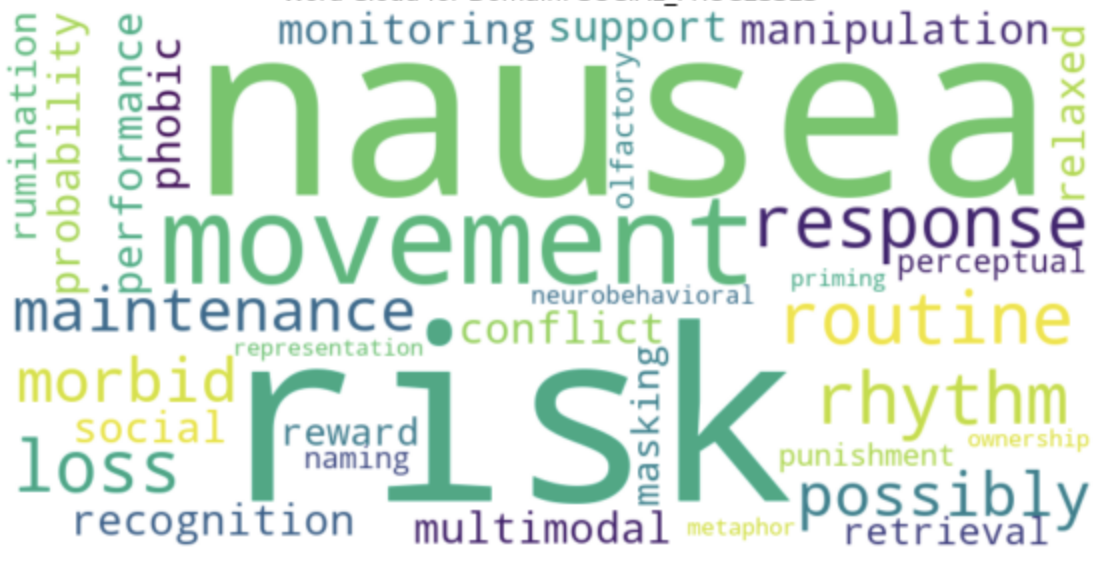

Supplement: Supplementary file 1 — Supplementary Material 1 [file 12911_2024_2554_MOESM1_ESM.docx]
